# *West*JEM Will No Longer Use the Term “Provider” to Refer to Physicians

**DOI:** 10.5811/westjem.2021.8.54452

**Published:** 2021-09-01

**Authors:** Andrew Phillips, Shahram Lotfipour, Mark I. Langdorf

**Affiliations:** * DHR Health, Departments of Emergency Medicine and Critical Care, Edinburg, Texas; † University of California, Irvine, Department of Emergency Medicine, Irvine, California; ‡ Eisenhower Health, Department of Emergency Medicine, Rancho Mirage, California

Increasingly, the lay and academic press has blurred the titles and roles of those who deliver various aspects of healthcare. This development confuses patients and fails to acknowledge the substantial differences in training and clinical experience.^1^

Therefore, beginning with the next issue, the *Western Journal of Emergency Medicine* will no longer publish the term “provider” in reference to physicians except as required to reference specific laws or formal program names. The decision to formally and publicly expunge a term from our written language should not be – and was not –taken lightly. Yet the evidence overwhelmingly supports the scientific and professional obligation of the *Journal* to accurately and respectfully refer to healthcare professionals of all degree types and roles. As we strive to phase out use of this term, we encourage other journals to do the same.

Medical journals must promote research that is clearly reported and replicable. Yet the term “provider” has no formal definition other than a person or entity who/that qualifies for payment from Medicare or Medicaid.^2^ It has been used in the literature to refer to institutions, physicians, physician assistants, nurse practitioners, emergency medical services personnel, midwives, dieticians, nurse anesthetists, pharmacists, and others. Research in manuscripts that use the term is neither clearly reported nor replicable across the differences in education, role and scope of the individuals. This is particularly important when reporting the sensitivity/specificity characteristics of diagnostic tests, especially involving operators with various levels of training. Point-of-care ultrasound is one such example.

Medical journals also report educational content that informs patient care. Thus, accurate and precise titles that reflect previous education are necessary. Use of the generic term “provider” when teaching medicine, nursing, physical therapy, and other healthcare facets blurs the composition of the medical team and its members. The term’s use may also contribute to postgraduate trainee burnout by devaluing both commitment to and duration of education.^3^

Spoken language in research and clinical settings evolves and is driven by the written word of medical journals.^4^ As a result, these journals bear a responsibility to foster appropriate, professional language. It is clear that many physicians dislike the term “provider.”^3^,^5^–^10^ Moreover, as a profession, multiple medical societies have formally called for removal of the term in reference to physicians,^11^–^15^ and medical journals should reflect such professional standards.

*WestJEM* is not the first medical journal to adopt this policy, but it has been at least 20 years since the first journal did, even as its use increases.^9^ A simple PubMed search showed that the term was used in more than 7000 peer-reviewed manuscripts in 2020 alone (personal search by AWP on June 18, 2021, for the term “provider” in all fields at https://pubmed.ncbi.nlm.nih.gov), the peak of an upward trend over the last decade. Medical journals worldwide must make a conscious decision to remove the word from manuscripts if the trend is to be reversed.

We hope that our formal commitment and rationale for this decision encourages other medical journals and authors to sunset the term “provider” in reference to physicians, and better clarify the roles of other clinicians in academic writing.
